# Novel approach for parallelizing pairwise comparison problems as applied to detecting segments identical by decent in whole-genome data

**DOI:** 10.1093/bioinformatics/btab084

**Published:** 2021-03-11

**Authors:** Emmanuel Sapin, Matthew C Keller

**Affiliations:** Institute for Behavioral Genetics, University of Colorado Boulder, Boulder, CO 80309, USA; Institute for Behavioral Genetics, University of Colorado Boulder, Boulder, CO 80309, USA; Psychology & Neuroscience Department, University of Colorado Boulder, Boulder, CO, USA

## Abstract

**Motivation:**

Pairwise comparison problems arise in many areas of science. In genomics, datasets are already large and getting larger, and so operations that require pairwise comparisons—either on pairs of SNPs or pairs of individuals—are extremely computationally challenging. We propose a generic algorithm for addressing pairwise comparison problems that breaks a large problem (of order *n*^2^ comparisons) into multiple smaller ones (each of order *n* comparisons), allowing for massive parallelization.

**Results:**

We demonstrated that this approach is very efficient for calling identical by descent (IBD) segments between all pairs of individuals in the UK Biobank dataset, with a 250-fold savings in time and 750-fold savings in memory over the standard approach to detecting such segments across the full dataset. This efficiency should extend to other methods of IBD calling and, more generally, to other pairwise comparison tasks in genomics or other areas of science.

**Availability and Implementation:**

A GitHub page is available at https://github.com/emmanuelsapin with the code to generate data needed for the implementation

## 1 Introduction

Situations where all pairs of observations in a dataset must be compared arise in many areas of science. For example, in protein studies, forming the graphs used in protein clustering relies on finding a protein’s likeness to every other protein (Chapman and Kalyanaraman, 2011; Sapin et al., 2016), and in physics, calculating the total force each body has on every other body is required in order to predict the position and motion of all bodies in the n-body problem (Leimanis and Minorsky, 1958). Such pairwise comparison problems are extremely computationally challenging with large datasets because they grow at the square of the sample size [are of order $O(n^{2})$].

Because genomics datasets are growing quickly in both numbers of samples and numbers of measured variants, the computational challenge inherent in comparing all pairs of observations is particularly acute in several types of genomic analyses, such as the detection of epistasis (Fang *et al.*, 2012; Sapin et al., 2014) and the construction of gene regulatory networks (Chang *et al.*, 2008; Qiu et al., 2009). Another such example in modern genomics, and the motivation for the approach introduced in this manuscript, is the detection of identical by descent (IBD) segments between all pairs of individuals using whole-genome single nucleotide polymorphism (SNP) data (Browning and Browning, 2012). Two haplotypes (homologous chromosomal segments of DNA) are IBD if they descend from a common ancestor without either haplotype experiencing an intervening recombination (Powell et al., 2010). IBD segments can be used for a number of downstream analyses in genetics, including imputation, phasing, inference of the degree of relatedness, IBD mapping to detect the effects of rare variants on phenotypes, estimation of the effective population sizes, and detection of signatures of recent positive selection (Gusev, 2011; Loh, 2016 ; Browning and Browning, 2012). IBD detection requires that each pair of individuals in a dataset is compared at each location across the genome, typically in phased data (where the homologous chromosomes inherited from the father and mother have been computationally distinguished from each other). Segments that match for a stretch that is too long to have arisen by chance are deemed ‘IBD,’ although both false positive (calling a segment IBD when it is not) and false negative (failing to detect an IBD segment) can occur, especially for shorter segments. This approach is usually parallelized by splitting the genome into 22 subsets corresponding to the 22 (non-sex) chromosomes, but further subsetting by smaller genomic (sub-chromosomal) windows is problematic because it increases the miss rate of IBD segments that span two or more windows, because it adds a computationally expensive post-processing step (stitching together IBD segments that span across windows) (Browning and Browning, 2012), and because each window still requires $O(n^{2})$ comparisons, which can become too computationally expensive in large datasets even if the windows are small.

Several methods have been proposed to compare all pairs in a dataset in efficient ways, although none of these have been applied to IBD detection to our knowledge. While some are applicable only to specific problems (Cormen *et al.*, 1990; Krejčí, 2018; Parikshit et al., 2009; Wauthier et al., 2013), a general strategy that can be applied across multiple applications is to parallelize the comparisons, hopefully in some kind of efficient manner. Kiefer et al. (Kiefer et al., 2010) reviewed three general approaches to accomplish this. The first (‘broadcast’) approach consists solely in having multiple processes performing comparisons each on a subset of pairs. The second (‘block’) approach subsets pairs that are more likely to have elements in common, which limits data replication [see also (Kleinheksel and Somani, 2016)]. The final (‘design’) approach uses a projective plane (Lee et al., 2004) or *symmetric balanced incomplete block design* to create subsets of elements such that any pair of elements is observed exactly once across all the subsets.

In the current manuscript, we introduce an approach for parallelizing the pairwise comparison problem, similarly to the ‘design’ approach in (Kiefer *et al.*, 2010), by subsetting the set of individuals such that each pair is observed exactly once across all the subsets. Here an affine plane (Hughes and Piper, 1973) is used for this purpose, creating slightly more subsets ($\approx n+\sqrt{n}$) than the *n* individuals in the set. The sample size for each subset ($\approx \sqrt{n}$) is roughly midway between the naive approach (2 individuals in each of n(n−1)/2 subsets) and the brute force approach (a single set of *n* individuals entailing n(n−1)/2 comparisons, excluding self-comparisons), which is an excellent middle ground for massive and efficient parallelization for many applications.

Applying our approach to the detection of IBD segments on genome-wide SNP data from 435, 187 individuals from the UK Biobank dataset (Bycroft *et al.*, 2018), we show that our approach resulted in a large saving in time over alternative approaches. Our approach should allow for efficient parallelization of IBD detection in much larger datasets than this one, and the general approach is easily extendable to other applications outside of IBD detection and genomics.

## 2 Parallelization methodology

**Fig. 1.**
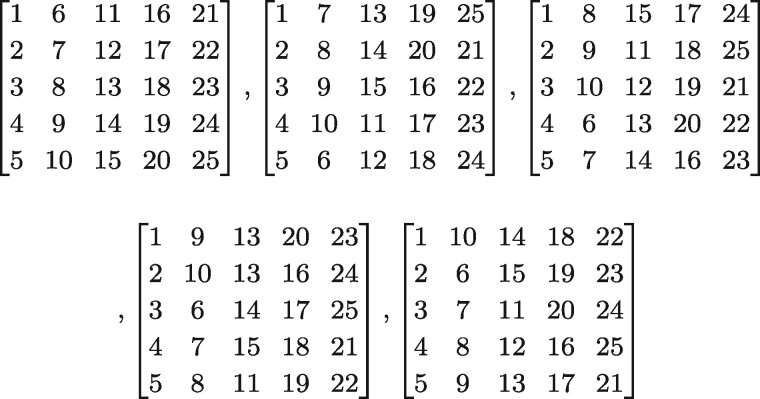
Matrix *P*, *P*_1_ and $P_{p-1}$

We describe here how to create subsets for samples of any size made up of individuals, *x_i_*, i∈ {1…n} with *n*≥ 4, such that all pairs of individuals, {*x_i_*, *x_j_*}, are observed exactly once across all subsets. Let *p* be the lowest prime number such that $p^{2}\geq n$. To create the subsets, the numbers from 1 to *p*^2^ are placed column-wise in a *p*-by-*p* matrix *P* as shown in Figure 1.Numbers 1…n index an individual in the sample, and numbers $n+1\ldots p^{2}$, if any, are unassociated (null), which can lead to some subsets having fewer individuals than others.

Our approach leads to a total of $p^{2}+p$ ($\approx n+\sqrt{n}$) unique subsets of individuals. The first *p* subsets are defined by each of the columns of matrix *P* and the next *p* subsets are defined by each of the rows of matrix *P*, for a total of 2*p* subsets drawn from matrix *P*. For example, the final subset based on the rows of *P* contains individuals *x_p_*, $x_{2p},\;x_{3p},\;\ldots x_{p^{2}}$.

The $p^{2}+p-2p=p^{2}-p$ remaining subsets are defined by new matrices, *P_k_*. Each matrix *P_k_* (k∈{1…p−1}) is created via shifting operations on the elements in the columns of *P*. Unlike the original 2*p* subsets based on *P*, subsets based on *P_k_* are defined only by each of the rows (not columns) of *P_k_*, and thus each *P_k_* matrix contributes *p* additional subsets. To create the *p—*1 *P_k_* matrices, the elements of each column *i* of *P_k_* are shifted up relative to the elements in the corresponding column *i* of *P* by *r*(*i*, *k*) rows, where

**Fig. 2.**
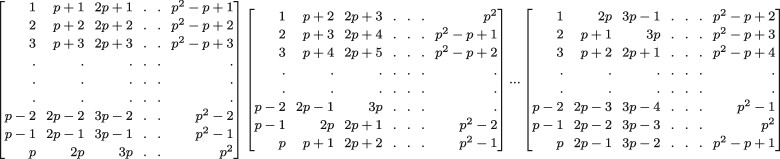
Matrices *P*, *P*_1_, *P*_2_, *P*_3_ and *P*_4_ for *n* = *p*^2^ = 25

(1) r(i,k)=((i−1)×k) mod p

and  mod  is the modulo operation. For example, for *k *=* *1, r(i=1,k=1)=0 and so the first column of *P*_1_ is identical to the first column of *P*, r(i=2,k=1)=1 and so the second column of *P*_1_ is shifted by one element relative to column 2 of *P*, and r(i=3,k=1)=2 and so the third column of *P*_1_ is shifted by two elements relative to column 3 of *P*, and so forth. This shifting is defined such that the elements of column *i* in *P* are shifted ‘downward’, in a conveyor belt type of motion, in the same column *i* in *P_k_*, as shown Figure 1. For example, the last element of the second column of *P*_1_ is the first element of the second column of *P* ($P_{1}[p,2]=P[1,2]$), and the last two elements of the third column of *P*_1_ are the first two elements of the third column of *P*. Thus, the total number of subsets is $p^{2}+p$ (of which 2*p* are created from the original matrix *P* and the remainder are created from shifted matrices *P_k_*), each individual is in *p *+* *1 different subsets, and the number of individuals per subset is *p* (or fewer for some subsets when $p^{2}>n$). Figure 2 shows the matrices *P*, *P*_1_, *P*_2_, *P*_3_ and *P*_4_ for an example where *n* = *p*^2^ = 25.

To assess this approach works as intended across a range of *n*, we counted the number of times *t* each pair of individuals was in the same subset across all subsets. As any pair should be in one and only one subset, *t* should be equal to 1 for all pairs, and this is what we observed for all n<5M. Although we have found no formal proof that this algorithm works to always define $p^{2}+p$ subsets such that each pair of individuals is in exactly one subset, we have computationally demonstrated that the algorithm works in this way for any n<5M and have no reason to suspect it would behave differently for n>5M. Therefore the collection of these subsets forms an affine plan where subsets are lines and individuals in a subset are points on the line.

Excluding self-comparisons, the total number of comparisons made under our approach is $\frac{p^{4}-p^{2}}{2}$, the product of the $p^{2}+p$ subsets and the $\frac{p^{2}-p}{2}$ comparisons per subset. The total number of comparisons under the naive approach, where all pairs are compared in the entire dataset, is $\frac{n^{2}-n}{2}$. Thus, under the optimal scenario where $p^{2}=n$, our approach requires exactly the same number of pairwise comparison computations as the naive approach but with the benefit of massive parallelization. Similarly, if self-comparisons are included, performing them only on the first *p* subsets would avoid multiple self-comparisons, again leading to the same number of comparisons as the naive approach. A GitHub page is available at https://github.com/emmanuelsapin with the code to generate lists of IDs of individuals composing each subset.

## 3 Computational performance of an implementation of the novel approach

Assuming no limits to number of tasks that can be run at the same time in parallel and that user time scales directly (1-to-1) with number of comparisons, our approach should reduce the time a pairwise comparison problem takes by a factor of *n*. This is because the novel approach makes $\approx \frac{p^{2}}{2}\approx \frac{n}{2}$ comparisons in a given subsample whereas the standard approach makes $\approx \frac{n^{2}}{2}$ comparisons in the full sample. Thus, with unlimited computing resources, calling IBD segments in the UKB would be >435K times faster using our approach than calling segments on the entire sample. However, the actual amount of time saved using our approach will rarely come close to this best-case scenario, and several real-world considerations will impact it. For one, there are overhead costs to parallelizing itself; for example, job schedulers can take time to allocate resources to a job, and start times for multiple jobs can be delayed compared to those for a single job. Because these costs do not typically scale directly with *n*, parallelizing can actually cause performance to be slower when *n* is small. For larger samples, when overhead is likely to be a small fraction of overall compute time, the degree to which our approach saves time depends heavily upon the type of tasks being performed and the computational architecture employed. For tasks that are CPU-bound, our approach is likely to result in a time savings proportionate to the number of cores that can be used in parallel at a given time. For tasks that are input/output (I/O)-bound, on the other hand, massive parallelization using this approach can generate a large number of competing I/O requests, which can slow down performance per job and lead to increasingly diminished returns (Ali *et al.*, 2009). The degree to which this occurs is a function of the I/O scheduling algorithm used by the operating system, the RAID configuration of the hard drives where the data is read/written, and performance attributes of the hard drive, such as solid-state (better) versus magnetic spinning, read/write speeds, and cache size. As we demonstrate below, depending on the task at hand and the computational architecture being used, it may be necessary for users to modify the code of existing pairwise comparison programs, especially with respect to I/O operations, in order to capitalize on the potential benefits of the novel approach described here.

To quantify the performance of our approach with respect to a particular pairwise comparison problem, we took subsamples of the UK Biobank data (Bycroft *et al.*, 2018), from *n *=* *100 up to the entire dataset (*n *=* *435, 187) and compared the time and RAM usage of the novel approach to the ‘standard’ approach for calling IBD segments across a dataset. We defined IBD segments as being >3.5 centimorgans in length, and called them using GERMLINE software ( Gusev et al., 2009), which is among the most efficient and accurate estimators of IBD (Bjelland et al., 2017). The standard approach ran GERMLINE on the entire sample but in parallel across the 22 chromosomes. Assuming that all 22 jobs could be run simultaneously, only the time and RAM usage for the largest (second) chromosome need be considered for calculating the performance of the standard approach. However, because the standard approach would have required more memory (2.4 TB of RAM) and time (nearly a year) than was possible on our system, we had to estimate its performance for the entire *n *=* *435, 187 sample via extrapolation from its performance in smaller subsamples.

To implement the novel parallelization approach for the purpose of detecting IBD segments, we split samples of different sizes into subsets of size *p* as described in Section 2 above and ran jobs of 1000 subsets each in parallel, with each job assigned to a single node. For example, on the full sample, the lowest prime number, *p*, such that $p^{2}\geq 435,187$ is 661, and thus *p *=* *661, resulting in $661^{2}+661=437,582$ subsets. Overall, therefore, this required 437, 582×22≈9.6M runs of GERMLINE for all pairs to be compared on all 22 chromosomes. We assigned 1000 of these instances of GERMLINE to each of 9627 jobs. Jobs, in turn, were assigned via the Slurm workload manager to one of 140 nodes in the Blanca Condo Cluster at the University of Colorado at Boulder (https://www.colorado.edu/rc/res-ources/blanca) and were run in parallel. Because we had GERMLINE extract the *p *=* *661 individuals defining each subset from the full $n\times m_{k}$ genotype file (where *m_k_* is the number of markers on chromosome *k*), unmodified GERMLINE would have attempted to access this large $n\times m_{k}$ file 1000 times per job, leading to ≈9.6M read operations overall. This large number of competing read requests would have dramatically slowed down each job and the overall performance of the novel approach. To avoid this, we modified GERMLINE to utilize shared memory segments. The $n\times m_{k}$ genotype file was read from the hard drive by a process only once per job and the memory segment this process generated was accessible to the 1000 instances of GERMLINE running on a given node thanks to a key number generated by commands in the C language using the *sysipc* library. This minor modification of GERMLINE reduced the total number of file read operations required by three orders of magnitude.

**Fig. 3.**
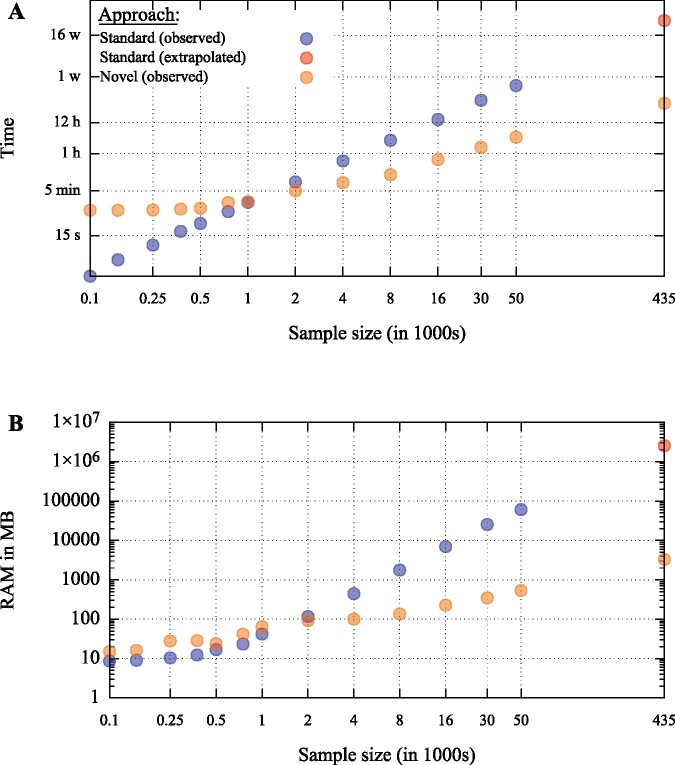
Comparison of time (**A**) and RAM (**B**) performance of the standard versus novel approach for calling IBD segments using GERMLINE (with parameters -min_m 3.5 -bits 75 -err-het 1 -err-hom 1 -w_extend) as a function of different sized subsamples of the UK Biobank. We assume all 22 chromosomes could be run in parallel using the standard approach, and so RAM and time results for this approach are for the longest (2nd) chromosome. The red points are linear extrapolations of the standard approach results on the log-log scale for *n *=* *435, 187

Figure 3 compares the time and RAM performance of the standard versus novel approaches to calling IBD segments as a function of sample sizes of random subsets drawn from the larger UK Biobank sample. Due to overhead costs to parallelization discussed above, the novel approach actually took longer than the standard approach when n≤1K. With increasing sample size, however, the novel approach led to an increasingly large speed advantage. For the full *n *=* *435, 187 sample, the final job using the novel approach ended 28.5 h after the first one was submitted (though 34/1000 jobs failed for technical reasons and were re-run) as compared to the estimated 7252 h (≈ 302 days) for the typical approach, a 254-fold savings in user time. Perhaps equally important, the novel approach required much less memory than the standard approach at larger sample sizes. For the full *n *=* *435, 187 sample, the novel approach used a maximum of ≈3.2 GB of RAM, whereas we predict that the standard approach would have used a maximum of 2.4 TB of RAM, a 750-fold savings in memory. Thus, using a novel approach, we were able to complete a large pairwise comparison problem that would have been impossible to run on our computational architecture using a standard approach. These results serve to illustrate the kind of performance improvements that can be achieved for pairwise comparison problems using this novel approach. The actual degree of improvement will, of course, depend on the specific problem at hand, but should be largely governed by the factors we have highlighted here.

## 4 Conclusion

We developed a novel parallelization strategy that subsets individuals from a larger sample in order to break a large pairwise comparison problem (of $O(n^{2})$) into multiple smaller pairwise comparisons problems (each of O(n)). Each pair of individuals is compared exactly once across all subsets and the total number of comparisons that must be made under our algorithm is the same as the number that must be made if all pairs were compared in the entire dataset without subsetting.

We demonstrated that this approach is very efficient for calling IBD segments using GERMLINE in the large UK Biobank dataset, with a 254-fold savings in time and 750-fold savings in memory over running GERMLINE on the entire sample in our particular instance. The IBD segments we obtained will be used to infer the degree of relatedness between all pairs of individuals in our dataset, which is another pairwise comparison problem that can be optimized using the same approach introduced here. The amount of time saved using our approach will depend on the particular algorithms being used and on the computational architecture, but to the degree that the algorithms scale over O(n), and especially as they approach $O(n^{2})$, as many algorithms that work at the unit of pairs should, our approach should offer substantial savings in user-end time and RAM. While our approach was highly efficient for calling IBD segments using GERMLINE, there are good reasons to believe that this efficiency will extend to other methods of IBD calling and, more generally, to other pairwise comparison tasks in genomics or other areas of science.
